# The miR‐511‐3p/ATP2A2 Axis Is Required for Propofol‐Mediated Cardioprotection Against Hypoxia/Reoxygenation Injury

**DOI:** 10.1155/cdr/7021184

**Published:** 2026-10-20

**Authors:** Yan Sun, Rongjuan Jiang, Bo Zhu, Xia Liu, Qian Li, Yu Huang, Shaoxing Liu

**Affiliations:** ^1^ West China School of Medicine Sichuan University Chengdu China scu.edu.cn; ^2^ Department of Anesthesiology Sichuan University Affiliated Chengdu Second People′s Hospital Chengdu China; ^3^ Department of Anesthesiology Chengdu Second People′s Hospital Chengdu China

**Keywords:** ATP2A2, inflammation, ischemia/reperfusion (I/R) injury, miR-511-3p, oxidative stress, propofol

## Abstract

**Background:**

Propofol (PPF) alleviates myocardial ischemia/reperfusion injury (MIRI), yet no existing studies have clarified the function of microRNA‐511‐3p (miR‐511‐3p) in cardiac injury or its link to PPF′s cardioprotection.

**Purpose:**

This study clarified how the miR‐511‐3p/ATPase sarcoplasmic/endoplasmic reticulum Ca^2+^ transporting 2 (ATP2A2) signaling cascade mediated PPF‐induced relief against hypoxia/reoxygenation (H/R) cardiac damage.

**Methods:**

H/R damage models were established in H9c2 cells. Cell viability was measured via the CCK‐8 assay, and flow cytometry was used to quantify the proportion of apoptotic cells. Cellular reactive oxygen species (ROS) levels were assessed using the DCFH‐DA fluorescent staining method. ELISA and colorimetric tests were combined to measure cardiac injury markers, inflammatory mediators, and oxidative stress‐related molecules. RT‐qPCR was applied to test the expression levels of miR‐511‐3p and ATP2A2. Dual‐luciferase reporter assays were carried out to confirm the direct binding interaction between the two molecules.

**Results:**

PPF markedly boosted the viability and restrained apoptosis of H9c2 cells subjected to H/R treatment. It also reduced the release of cardiac injury markers and eased excessive oxidative stress and inflammatory responses. H/R exposure raised miR‐511‐3p abundance, an alteration that PPF treatment could reverse. Forced miR‐511‐3p expression partially abolished the cardioprotective benefits brought by PPF. ATP2A2 was verified as a direct target of miR‐511‐3p, whose expression was suppressed by this miRNA. Upregulating ATP2A2 counteracted cell damage triggered by miR‐511‐3p overexpression.

**Conclusions:**

PPF might mitigate H/R‐triggered cardiomyocyte damage, possibly through inhibiting miR‐511‐3p and increasing its target ATP2A2, and this molecular axis may offer a tentative therapeutic reference for MIRI.

## 1. Introduction

Ischemic heart disease (IHD) acts as a key driver of rising morbidity, impaired life quality, amplified medical resource expenditure, and aggravated cardiovascular episodes [1]. Restoring blood perfusion to ischemic tissues serves as an effective clinical strategy to save patients, yet this reperfusion procedure can trigger secondary cardiac damage termed myocardial ischemia/reperfusion injury (MIRI) [2]. MIRI further impairs cardiac performance by triggering arrhythmia, myocardial infarction, and even sudden cardiac death [3], and it stands as a major risk factor for high mortality rates following myocardial infarction [4]. The hypoxia/reoxygenation (H/R) model has been widely adopted as an in vitro experimental tool to probe the mechanisms underlying I/R injury [5, 6].

As a widely applied intravenous anesthetic in clinical practice, propofol (PPF) boasts favorable pharmacological traits, including rapid induction, fast recovery, and low risks of postoperative nausea and vomiting [7]. Beyond its anesthetic potency, this agent exerts diverse nonanesthetic bioactivities [8]. Multiple preclinical animal studies have indicated that PPF has the capacity to ease MIRI; it delivers cardioprotective outcomes by alleviating cardiac tissue lesions, shrinking infarct volume, and restoring cardiac function [9]. Cellular research further validated that PPF could suppress oxidative stress, inflammatory response, and cardiomyocyte apoptosis to relieve damage elicited by MIRI [10, 11]. Recent studies proposed that such protective phenotypes might be modulated by small noncoding RNAs, microRNAs (miRNAs), for instance [12]. Several prior works have analyzed the expression shifts and biological functions of miRNAs during PPF‐mediated cardiac protection. One relevant study revealed that PPF mitigated cardiomyocyte I/R damage by modulating the MALAT1/miR‐206/ATG3 molecular cascade [13]. Meanwhile, bioinformatics profiling detected higher miR‐511‐3p abundance in cardiomyocytes following damage onset in rat MIRI models [14]. Nevertheless, the detailed biological functions and molecular mechanisms of this miRNA in cardiac injury remain unclear, and it also remains unclear whether this miRNA participates in PPF‐induced cardiac protection.

ATPase sarcoplasmic/endoplasmic reticulum Ca^2+^ transporting 2 (ATP2A2) encodes the endoplasmic reticulum transmembrane protein SERCA2 [15], and abundant research has validated this molecule′s regulatory role in cardiac injury. Oxygen free radicals trigger membrane lipid peroxidation to raise the permeability of cardiomyocyte membranes; these radicals also directly target SERCA2 on the sarcoplasmic reticulum, weaken its performance or suppress its activity, and ultimately provoke calcium overload [16]. Existing mechanistic investigations indicated that ATP2A2 knockdown counteracts the cardioprotective phenotypes induced by miR‐429, whereas circ_0004771 alleviates cardiac damage through the miR‐429/ATP2A2 molecular axis [17]. Part of the cardiac protective effects from matrix metalloproteinase inhibitors stems from slowed degradation of SERCA2a protein [18]. Ischemic stress elevates serine 663 phosphorylation of SERCA2 in cardiac tissues, whereas inhibition of this specific phosphorylation rescues SERCA2 calcium transport function and thereby exerts cardiomyocyte protection [19].

Given the above research context, this work is aimed at exploring the function of miR‐511‐3p in PPF‐mediated relief against cardiomyocyte H/R injury, identifying whether this miRNA shapes cardiac damage via targeted regulation of ATP2A2, and uncovering novel candidate intervention targets for the prevention and management of MIRI.

## 2. Materials and Methods

### 2.1. Cell Culture

The embryonic rat cardiomyocyte H9c2 cell line was purchased from Procell (China). Cells used in all experiments were maintained within passages 3–8 to ensure phenotypic stability; mycoplasma contamination was routinely tested and excluded before use. Cells were cultured in basal DMEM medium (Gibco, United States) supplemented with 10% fetal bovine serum (Gibco, United States) and 1% penicillin‐streptomycin double‐antibiotic solution (Hyclone, United States). To establish an in vitro H/R model mimicking MIRI, cells were shifted to a tri‐gas hypoxic incubator (BioSpherix, United States) and incubated under low‐oxygen settings (1% O₂, 95% N₂, and 5% CO₂) for 12 h, followed by 6 h of reoxygenation under standard culture conditions [20]. Cells assigned to the control group remained under normoxic culture throughout the whole process.

Before hypoxic incubation, the culture medium was replaced with fresh complete medium containing PPF (Sigma‐Aldrich, United States). Four PPF concentrations (0, 25, 50, and 100 *μ*mol/L) were set for separate intervention groups.

### 2.2. Cell Transfection

Cell transfection was carried out using Lipofectamine 3000 transfection reagent (Invitrogen, United States). MiR‐511‐3p mimics, ATP2A2 overexpression plasmids, and corresponding negative control sequences were all synthesized by GenePharma (China). H9c2 cells were seeded 24 h before transfection until the cell confluence reached approximately 70%. Briefly, plasmid DNA and Lipofectamine 3000 were separately diluted in serum‐free DMEM, combined at the manufacturer‐recommended ratio, and incubated at room temperature for 15 min to form transfection complexes. The mixture was then added dropwise to the cell culture medium. After 6 h of incubation in a CO₂ incubator, the medium was replaced with fresh complete medium. Cells were cultured for an additional 48 h before harvest for subsequent assays.

### 2.3. Cell Counting Kit‐8 (CCK‐8)

Cell viability of H9c2 cells was examined with the CCK‐8 cell activity detection kit (Beyotime, China). After seeding cells in 96‐well plates and finishing corresponding experimental treatments, CCK‐8 working solution was supplemented into each well at four time points (0, 24, 48, and 72 h), followed by incubation without light. A multimode microplate reader (BioTek, United States) was then used to record absorbance values at the 450‐nm wavelength.

### 2.4. Flow Cytometry

Apoptotic rates of H9c2 cells were quantified using the Annexin V‐FITC/PI apoptosis assay kit (BD, United States). Cells harvested from all groups were washed and resuspended, incubated with Annexin V‐FITC and PI staining reagents successively in the dark, and measured on a BD flow cytometer to calculate the percentage of apoptotic cells.

### 2.5. Cysteine‐Aspartic Acid Protease 3 (Caspase‐3) Activity Measurement

Caspase‐3 enzymatic activity in H9c2 cells was quantified with a Caspase‐3 activity assay kit (Cell Signaling Technology, United States). Cells from all groups were fully lysed in lysis buffer, and supernatants were isolated after centrifugation. The substrate Ac‐DEVD‐pNA was mixed with supernatants and incubated at 37°C in the dark for 2 h. Absorbance signals at 405 nm were read via a multimode microplate reader (BioTek, United States) to reflect the activation degree of Caspase‐3.

### 2.6. Colorimetric Assays for Enzyme Activity

Colorimetric quantitative analysis was performed with lactate dehydrogenase (LDH) assay kits (Abcam, United Kingdom) and malondialdehyde (MDA) detection kits (Elabscience, China). Cell culture supernatants were collected, and color‐developing incubation steps were carried out in strict accordance with the manufacturer′s instructions. Absorbance values at matched wavelengths were measured by a multimode microplate reader (BioTek, United States), and LDH activity alongside MDA contents were calculated using standard calibration curves.

### 2.7. Enzyme‐Linked Immunosorbent Assay (ELISA)

ELISA kits for rat creatine kinase‐MB (CK‐MB), heart‐type fatty acid‐binding protein (H‐FABP), tumor necrosis factor‐*α* (TNF‐*α*), interleukin‐6 (IL‐6), and interleukin‐1*β* (IL‐1*β*) were purchased from Elabscience (China), and the rat superoxide dismutase (SOD) kit was sourced from Jianglai Biotechnology (China). Cell supernatants of each group were collected and centrifuged to remove sediments. Prepared supernatants and serially diluted standards were loaded into microplates and incubated at a constant temperature to facilitate antigen–antibody binding. After washing away unbound substances, enzyme conjugates were supplemented for secondary incubation, followed by another wash procedure. Color development was initiated with chromogenic substrates under dark conditions and terminated using the stop solution. Well absorbance was measured at specific wavelengths with a BioTek multimode microplate reader. Standard curves were established based on standard samples, and the protein levels of all test biomarkers in each group were quantified correspondingly.

### 2.8. 2 ^′^,7 ^′^‐Dichlorodihydrofluorescein Diacetate (DCFH‐DA) Fluorescence Assay

The cellular level of reactive oxygen species (ROS) was determined using a DCFH‐DA ROS detection kit (Beyotime, China). After discarding the original culture medium, cells were incubated with the prepared DCFH‐DA working solution under dark conditions. Following cell rinsing, fluorescence intensity was quantified via a multimode microplate reader (BioTek, United States), which was applied to evaluate the relative intracellular ROS content.

### 2.9. Reverse Transcription Quantitative Real‐Time Polymerase Chain Reaction (RT‐qPCR)

Total RNA was isolated from H9c2 cells of all groups using TRIzol reagent (Invitrogen, United States). Purified RNA was reverse‐transcribed into cDNA with a reverse transcription kit (Takara, Japan) following official protocols. RT‐qPCR amplification was carried out on an Applied Biosystems real‐time thermal cycler with SYBR Green RT‐qPCR reagents (Takara, Japan). U6 and GAPDH served as reference genes for miRNA and mRNA detection separately. Primers used for RT‐qPCR were synthesized by Sangon Biotech (Shanghai, China). The primer sequences were provided in Table S1. The 2^−*ΔΔ*Ct^ approach was adopted to compute the relative expression abundance of target genes.

### 2.10. Identification of Downstream Target Genes of miR‐511‐3p

Two online datasets, TargetScan (https://www.targetscan.org/vert_80/) and miRDB (https://mirdb.org/), were utilized to screen potential downstream genes regulated by miR‐511‐3p. The GeneCards database (http://www.genecards.org) was queried to collect candidate genes linked to cardiac injury, and entries with relevance scores below 100 were discarded due to insufficient supporting evidence connecting them to myocardial damage. Overlapping genes shared across the three datasets were extracted as candidate targets of miR‐511‐3p for subsequent experimental validation.

### 2.11. Dual Luciferase Reporter Gene Assay

Dual‐luciferase reporter plasmids carrying wild‐type and mutant ATP2A2 sequences were synthesized by GenePharma (China). These reporter constructs were cotransfected into H9c2 cells alongside miR‐511‐3p mimics with Lipofectamine 3000 transfection reagent (Invitrogen, United States). Forty‐eight hours post‐transfection, cells were lysed using a dual‐luciferase assay kit (Promega, United States). Luminescence signals of firefly luciferase and Renilla luciferase were sequentially measured on a multimode microplate reader (BioTek, United States). Relative luciferase activity was normalized to Renilla luciferase readouts to verify the direct binding interaction between miR‐511‐3p and ATP2A2.

### 2.12. Statistical Analysis

All assays in this study were performed with three independent biological replicates. Measurement data were expressed as mean ± standard deviation. Comparisons between two groups were analyzed via an independent‐samples *t*‐test. One‐way analysis of variance (ANOVA) or two‐way ANOVA was applied to evaluate statistical differences among multiple groups. All statistical computations were processed using GraphPad Prism 9.3.1 software. A *p* value less than 0.05 was defined as the threshold for statistically significant differences.

## 3. Results

### 3.1. Regulatory Effects of PPF on Viability, Apoptosis, and Injury Markers in H/R‐Stimulated Cardiomyocytes

H/R exposure markedly suppressed the viability of H9c2 cells. Supplemental treatment with serially diluted PPF restored cell viability to varying degrees, with the 50 *μ*mol/L group showing a marked protective effect (*p* < 0.001). No statistical disparity in cell viability was observed between the 50 and 100 *μ*mol/L groups, so 50 *μ*mol/L PPF was adopted as the intervention concentration for all subsequent assays (Figure 1A).

**Figure 1:**
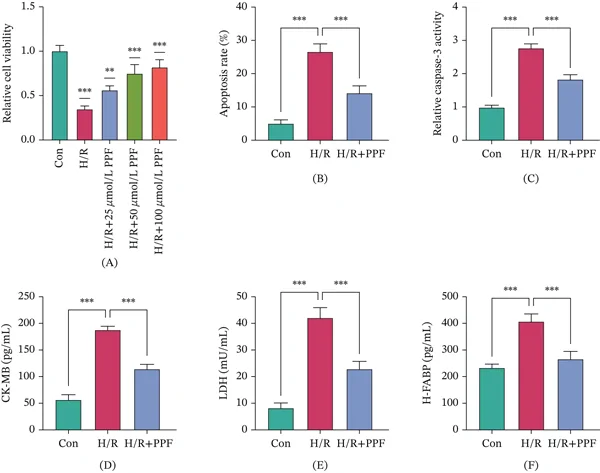
Effects of propofol treatment on cell viability, apoptosis, and injury biomarkers in hypoxia/reoxygenation (H/R)‐injured cardiomyocytes. (A) Cell viability of H9c2 cells treated with gradient concentrations of propofol (0, 25, 50, and 100 *μ*mol/L). (B) Apoptosis levels of H9c2 cells in each group detected by flow cytometry. (C) Colorimetric measurement of Caspase‐3 enzymatic activity in cell lysates of each group. (D) ELISA detection of creatine kinase‐MB (CK‐MB) protein concentrations in cell culture supernatant of each group. (E) Colorimetric kit assay of lactate dehydrogenase (LDH) activity in cell culture supernatant of each group. (F) ELISA measurement of heart‐type fatty acid‐binding protein (H‐FABP) levels in cell culture supernatant of each group. ∗∗*p* < 0.01; ∗∗∗*p* < 0.001.

Apoptosis measurement revealed that H/R exposure greatly raised the apoptotic ratio, whereas cotreatment with PPF reversed this adverse outcome (Figure 1B). Assays measuring Caspase‐3 enzymatic activity further validated the above apoptotic alterations: H/R treatment boosted Caspase‐3 activity, and PPF co‐incubation blocked such activation (Figure 1C).

For myocardial injury indicators, H/R stimulation elevated supernatant levels of CK‐MB, LDH, and H‐FABP simultaneously, which indicated compromised cell membrane integrity. Co‐administration of PPF reduced the release of all three injury markers and alleviated H/R‐triggered cellular damage in cardiomyocytes (Figure 1D–F).

### 3.2. Modulation of PPF on H/R‐Triggered Oxidative Stress and Inflammatory Response in Cardiomyocytes

Previous reports have proven that oxidative stress and inflammatory responses jointly exacerbate cardiomyocyte injury and impair cardiac function [21, 22]. This work further explored whether PPF could regulate oxidative and inflammatory phenotypes in H/R‐challenged cardiomyocytes. Detection data demonstrated that H/R treatment elevated intracellular ROS and MDA levels while lowering SOD abundance. PPF supplementation reversed these abnormal variations and mitigated oxidative stress damage (Figure 2A–C).

**Figure 2:**
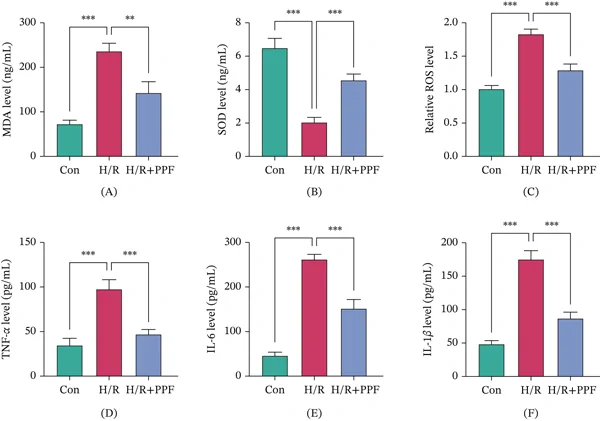
Effects of propofol intervention on oxidative stress and inflammatory factors in H/R‐stimulated cardiomyocytes. (A) Intracellular malondialdehyde (MDA) levels in H9c2 cells of each group determined by colorimetric kits. (B) ELISA detection of superoxide dismutase (SOD) expression in cells of each group. (C) Relative reactive oxygen species (ROS) contents in cells of each group measured using fluorescent probes. (D) ELISA quantification of tumor necrosis factor‐*α* (TNF‐*α*) concentrations in cell culture supernatant of each group. (E) ELISA detection of interleukin‐6 (IL‐6) concentrations in cell culture supernatant of each group. (F) ELISA measurement of interleukin‐1*β* (IL‐1*β*) concentrations in cell culture supernatant of each group. ∗∗*p* < 0.01; ∗∗∗*p* < 0.001.

Measurements of inflammatory cytokines in culture supernatants showed that H/R stimulation boosted the secretion of TNF‐*α*, IL‐6, and IL‐1*β*. Coculture with PPF suppressed the release of all three pro‐inflammatory mediators and relieved cellular inflammatory activation (Figure 2D–F).

### 3.3. Overexpression of miR‐511‐3p Attenuated PPF‐Mediated Relief of H/R‐Induced Viability Loss, Apoptosis, and Myocardial Injury

RT‐qPCR results illustrated that H/R treatment elevated miR‐511‐3p abundance in H9c2 cells, whereas PPF incubation markedly suppressed its expression. Transfection efficiency of the miR‐511‐3p overexpression construct was also validated in parallel (Figure 3A). CCK‐8 readings indicated that forced miR‐511‐3p expression weakened cellular viability (Figure 3B). Flow cytometry and apoptosis‐related biochemical tests revealed simultaneous increases in apoptotic percentage and Caspase‐3 enzymatic activity within the miR‐511‐3p overexpression group (Figure 3C–D). Assays for myocardial injury markers further confirmed that miR‐511‐3p overexpression raised supernatant concentrations of CK‐MB, LDH, and H‐FABP (Figure 3E–G).

**Figure 3:**
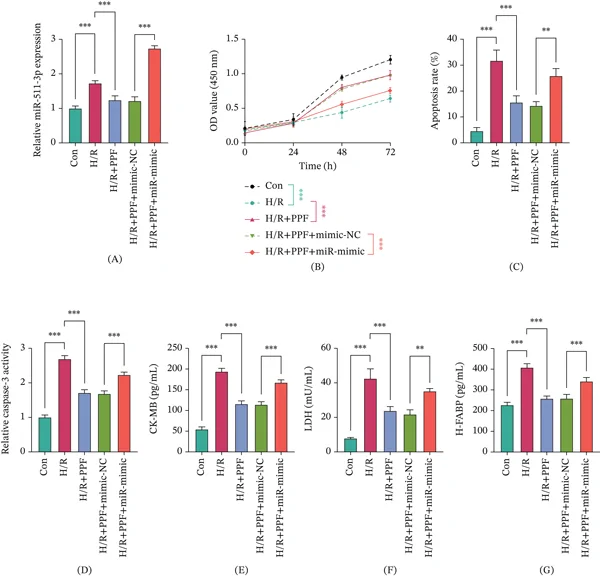
Detection of the reversal effect of miR‐511‐3p on propofol‐mediated cardioprotection. (A) RT‐qPCR detection of miR‐511‐3p expression after propofol treatment and verification of transfection efficiency of miR‐511‐3p overexpression plasmid. (B) Cell viability of H9c2 cells in each group assessed by CCK‐8 assay. (C) Apoptosis levels of cells in each group analyzed via flow cytometry. (D) Colorimetric kit detection of cellular Caspase‐3 enzymatic activity in each group. (E) ELISA measurement of CK‐MB protein content in cell supernatant. (F) Colorimetric kit assay of LDH activity in cell supernatant. (G) ELISA quantification of H‐FABP protein levels in cell supernatant. ∗∗*p* < 0.01; ∗∗∗*p* < 0.001.

Collectively, these data suggested that miR‐511‐3p overexpression mitigated the protective influence of PPF against H/R damage, reducing cell survival and aggravating apoptosis and cellular injury.

### 3.4. Overexpression of miR‐511‐3p Blunted PPF‐Induced Alleviation of H/R‐Triggered Oxidative Stress and Inflammation

Measurements of oxidative stress biomarkers revealed that miR‐511‐3p overexpression further raised intracellular ROS and MDA levels while reducing SOD content (Figure 4A–C). ELISA quantification of inflammatory cytokines showed that miR‐511‐3p overexpression markedly boosted the secretion of TNF‐*α*, IL‐6, and IL‐1*β* (Figure 4D–F).

**Figure 4:**
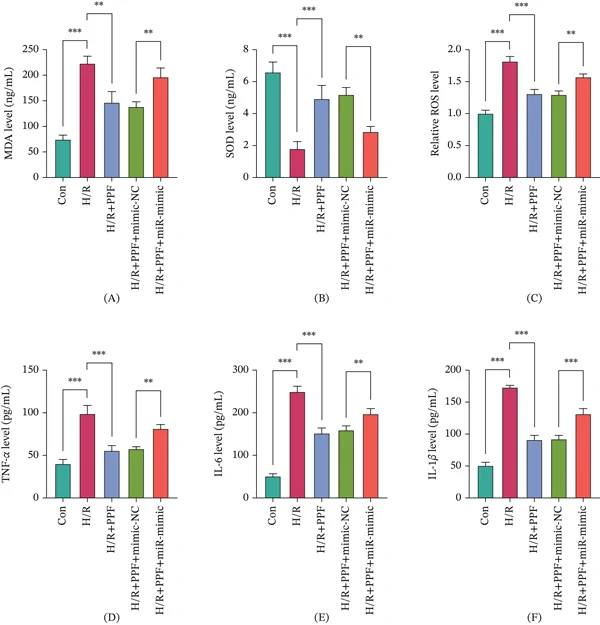
Analysis of the antagonistic effect of miR‐511‐3p overexpression on propofol‐alleviated H/R‐triggered oxidative stress and inflammation. (A) MDA content in H9c2 cells of each group detected by commercial kits. (B) ELISA detection of cellular SOD levels in each group. (C) Relative intracellular ROS contents of each group determined by fluorescent probe staining. (D) ELISA measurement of TNF‐*α* concentrations in cell culture supernatant. (E) ELISA quantification of IL‐6 concentrations in cell culture supernatant. (F) ELISA detection of IL‐1*β* concentrations in cell culture supernatant. ∗∗*p* < 0.01; ∗∗∗*p* < 0.001.

These observations indicated that miR‐511‐3p overexpression diminished the protective effects of PPF and exacerbated H/R‐elicited oxidative stress and inflammatory responses in H9c2 cells.

### 3.5. Target Binding and Regulatory Effect of miR‐511‐3p on ATP2A2

Five overlapping target genes were obtained from the intersection of three databases. After cross‐referencing published studies, ATP2A2 was chosen for subsequent mechanistic validation due to its frequent involvement in myocardial injury and high predicted binding affinity with miR‐511‐3p (Figure 5A). TargetScan prediction uncovered a miR‐511‐3p binding site within the ATP2A2 3 ^′^UTR, with a Context++ score of −0.24 and a Context++ score percentile of 95, indicating reliable and relatively strong binding (Figure 5B).

**Figure 5:**
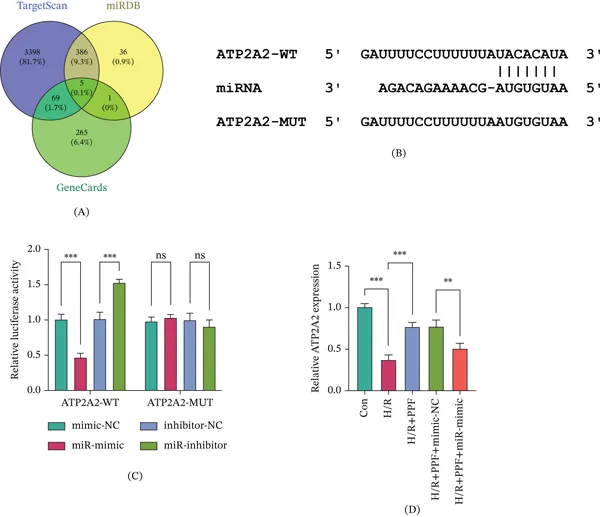
Prediction and experimental validation of ATP2A2 as a direct target of miR‐511‐3p. (A) Venn diagram showing candidate target genes screened by intersecting results from three target prediction databases. (B) Binding sequences and site score information of miR‐511‐3p with the 3 ^′^UTR of ATP2A2 predicted by the TargetScan database. (C) Dual‐luciferase reporter assay verifying the direct binding between miR‐511‐3p and ATP2A2. (D) RT‐qPCR detection of ATP2A2 mRNA expression in H9c2 cells under different treatments. ∗∗*p* < 0.01; ∗∗∗*p* < 0.001; ns, not significant.

Dual‐luciferase reporter assays validated their direct interaction. Co‐transfection with miR‐511‐3p mimics lowered luciferase activity, whereas miR‐511‐3p inhibitors increased this value. No obvious changes in luciferase activity were observed for the mutant reporter construct group (Figure 5C). RT‐qPCR data showed that H/R treatment reduced ATP2A2 transcription in H9c2 cells. PPF reversed this suppression, yet miR‐511‐3p overexpression once again downregulated ATP2A2 levels (Figure 5D).

### 3.6. Overexpression of ATP2A2 Blunted miR‐511‐3p‐Triggered Viability Loss, Apoptosis, and Myocardial Injury

RT‐qPCR was first applied to confirm the transfection efficiency of the ATP2A2 overexpression vector (Figure 6A). CCK‐8 assays revealed that co‐transfection with ATP2A2 overexpression plasmids restored cell viability and partially counteracted the suppressive influence of miR‐511‐3p on cell survival (Figure 6B). Assays for apoptotic indicators demonstrated that elevated ATP2A2 reduced apoptotic rates and Caspase‐3 activity, relieving miR‐511‐3p‐evoked apoptosis (Figure 6C–D). Measurements of myocardial injury markers showed that ATP2A2 overexpression cut down supernatant CK‐MB, LDH, and H‐FABP levels and alleviated miR‐511‐3p‐mediated cellular damage (Figure 6E–G).

**Figure 6:**
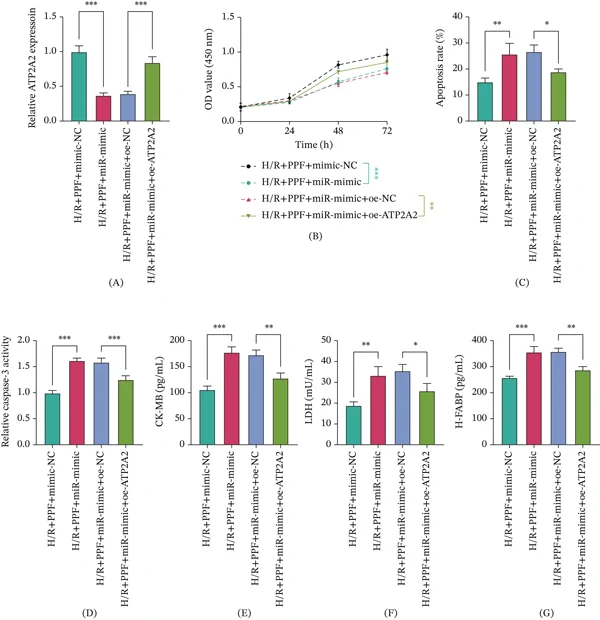
Functional verification of ATP2A2 overexpression in rescuing miR‐511‐3p‐mediated cardiomyocyte injury under H/R stimulation. (A) RT‐qPCR verification of the transfection efficiency of the ATP2A2 overexpression plasmid. (B) Viability of H9c2 cardiomyocytes in each group measured by CCK‐8 assay. (C) Quantitative analysis of cellular apoptosis levels in each group via flow cytometry. (D) Colorimetric kit detection of Caspase‐3 enzymatic activity in cells of each group. (E) ELISA measurement of CK‐MB protein content in cell culture supernatant. (F) Colorimetric kit assay of LDH activity in cell culture supernatant. (G) ELISA quantification of H‐FABP protein levels in cell culture supernatant. ∗*p* < 0.05; ∗∗*p* < 0.01; ∗∗∗*p* < 0.001.

These results indicated that upregulating ATP2A2 reversed miR‐511‐3p‐induced cardiomyocyte injury, improved cell viability, and restrained apoptosis and cellular damage.

### 3.7. Overexpression of ATP2A2 Suppressed miR‐511‐3p‐Mediated Oxidative Stress and Inflammatory Activation

Oxidative stress measurements showed that ATP2A2 upregulation lowered intracellular MDA and ROS while elevating SOD levels, alleviating miR‐511‐3p‐triggered oxidative stress (Figure 7A–C). ELISA data demonstrated that ATP2A2 overexpression reduced the secretion of pro‐inflammatory TNF‐*α*, IL‐6, and IL‐1*β*, and restrained miR‐511‐3p‐induced inflammatory activation (Figure 7D–F).

**Figure 7:**
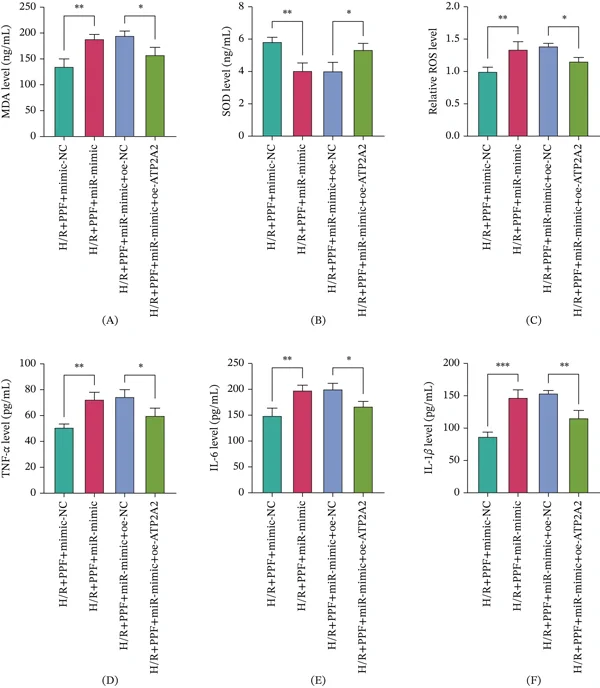
Effects of ATP2A2 overexpression on miR‐511‐3p‐triggered oxidative stress and inflammatory activation. (A) Intracellular MDA contents in H9c2 cells of each group detected using detection kits. (B) ELISA measurement of cellular SOD levels in each group. (C) Relative intracellular ROS contents of each group tested by fluorescent probes. (D) ELISA quantification of TNF‐*α* protein concentrations in cell culture supernatant. (E) ELISA detection of IL‐6 protein concentrations in cell culture supernatant. (F) ELISA measurement of IL‐1*β* protein concentrations in cell culture supernatant. ∗*p* < 0.05; ∗∗*p* < 0.01; ∗∗∗*p* < 0.001.

Collectively, elevated ATP2A2 partially alleviated miR‐511‐3p‐induced cellular damage and mitigated excessive oxidative stress and inflammatory responses.

## 4. Discussion

Previous work validated the cardioprotective capacity of PPF [23]. This study further illustrated a molecular cascade underlying PPF‐mediated cardiac protection. An in vitro H/R injury model was established using H9c2 cardiomyocytes. Our data revealed that PPF substantially relieved H/R‐elicited cellular damage, improved cell survival, and restrained apoptosis, the release of cardiac injury biomarkers, and oxidative stress. Such protective effects of PPF appeared tightly linked to the regulatory interplay between miR‐511‐3p and its downstream target ATP2A2.

Myocardial injury is driven by multiple overlapping pathological cascades. MIRI activates the mitochondrial apoptotic pathway, triggers cytochrome C release, and initiates caspase cascades to ultimately induce cardiomyocyte programmed death [24]. MIRI also disrupts cardiomyocyte membrane integrity and leads to massive leakage of intracellular CK‐MB, LDH, and H‐FABP. Circulating levels of these biomarkers directly reflect the severity of cardiac damage; H‐FABP can be detected within one hour of acute myocardial infarction onset and delivers superior sensitivity for early diagnosis [25]. Massive ROS accumulate during reperfusion and provoke oxidative stress, which further activates the ALOX15‐dependent phospholipid peroxidation axis and amplifies inflammatory cascades to aggravate secondary injury of viable cardiomyocytes in the infarct border zone [26]. These injury pathways interconnect to form an integrated pathophysiological network of cardiac damage, and interrupting this chain reaction represents a core therapeutic strategy against MIRI. Based on these pathological mechanisms, this study examined cell viability, apoptosis, injury biomarkers, oxidative stress, and inflammation. Our data showed that PPF treatment improved the viability of H/R‐exposed H9c2 cells, suppressed apoptotic progression, reduced CK‐MB, LDH, and H‐FABP release, and lowered levels of oxidative stress indicators and pro‐inflammatory cytokines. Consistent with previous relevant observations [27], these findings verified the prominent in vitro cardioprotective property of PPF and indicated that PPF alleviated cardiomyocyte damage via concurrent suppression of multiple injury pathways.

MiR‐511‐3p has been studied in multiple diseases and functions as a damage‐promoting factor. For instance, miR‐511‐3p was upregulated in liver tissues of hepatic sinusoidal obstruction syndrome (HSOS) rat models and monocrotaline‐treated liver sinusoidal endothelial cells, and aggravated HSOS by targeting Ptch1 to trigger Hedgehog signaling [28]. Significant elevation of miR‐511‐3p was also detected in renal interstitial fibrosis models. Excessive miR‐511‐3p repressed CAMK2N1 and boosted fibrosis‐related proteins to accelerate fibrotic progression [29]. Moreover, circulating miR‐511‐3p levels were markedly higher in children with congenital heart defects than in healthy identical twin controls, consistent with microarray and RT‐qPCR data, implying the involvement of this miRNA in congenital heart disorder pathogenesis [30]. Our experimental findings aligned with these prior reports: H/R stimulation raised miR‐511‐3p levels in H9c2 cells, and artificial overexpression of this miRNA further worsened cardiomyocyte injury. Nevertheless, miR‐511‐3p exhibited reduced expression and exerted protective functions in disorders such as stroke [31] and breast cancer [32]. Combining published records and our present results, miR‐511‐3p displayed obvious tissue‐ and disease‐specific biological functions. It functioned as an injury‐promoting factor under certain pathological conditions. Its upregulation aggravated cellular damage in such contexts, and targeted suppression of miR‐511‐3p might serve as a candidate therapeutic strategy for related tissue injuries.

This work further explored downstream regulatory cascades of miR‐511‐3p and validated ATP2A2 as its direct target gene, negatively modulated by this miRNA. H/R exposure markedly suppressed ATP2A2 expression in cardiomyocytes, whereas forced ATP2A2 upregulation effectively counteracted damage induced by miR‐511‐3p and exerted cardioprotective influences, consistent with outcomes from multiple previous investigations. Earlier studies reported that MIRI suppressed Sp1 expression and thereby downregulated SERCA2a. Luteolin elevated Sp1 activity to boost SERCA2a transcription and mitigate MIRI [33]. Genetic manipulation or pharmaceutical interventions that raised SERCA2a levels restored cardiac function and stabilized myocardial metabolic balance in diabetic mice [34]. Other research also indicated that the cardioprotective capacity of PPF relied on ATP2A2. Hepatic I/R treatment lowered SERCA2 mRNA and protein abundance in myocardium, and PPF incubation restored SERCA2 expression in rat cardiac tissue while restraining secondary cardiomyocyte apoptosis triggered by hepatic I/R [35]. Collectively, published evidence and our experimental data demonstrated that SERCA2, encoded by ATP2A2, was generally downregulated across diverse myocardial injury models and acted as a protective factor sustaining cardiomyocyte homeostasis.

However, the present study extends previous findings in several important aspects. Prior studies on PPF‐induced cardioprotection have primarily focused on its downstream protective phenotypes, such as reduced apoptosis, oxidative stress, and inflammation, or restoration of SERCA2a/ATP2A2 expression [20, 35, 36]. Nevertheless, the upstream mechanism linking PPF to ATP2A2 under H/R conditions has remained unclear. In the present study, we identified miR‐511‐3p as an H/R‐induced injury‐promoting miRNA in H9c2 cardiomyocytes, verified ATP2A2 as its direct target, and demonstrated that PPF protects cardiomyocytes by downregulating miR‐511‐3p, thereby restoring ATP2A2 expression. This miR‐511‐3p/ATP2A2 axis at least partially explains how PPF simultaneously suppresses apoptosis, oxidative stress, inflammatory cytokine production, and the release of cardiac injury biomarkers. Thus, the novelty of this work lies not only in confirming the cardioprotective effect of PPF, but also in defining a new upstream miRNA‐mediated mechanism and a potential therapeutic target. These findings complement previous studies on the cardioprotective mechanisms of PPF and provide a reference for future research on the miR‐511‐3p/ATP2A2 axis.

Beyond the mechanistic findings, it is necessary to critically evaluate the suitability of the H9c2 cardiomyoblast cell line as the experimental model in the present study. H9c2 cardiomyoblasts, an immortalized rat embryonic cardiomyocyte‐derived cell line, represent a widely adopted in vitro platform for investigating myocardial H/R injury [37, 38]. Its major strengths include easy maintenance, stable proliferation, and suitability for high‐throughput genetic manipulation such as miRNA overexpression/silencing and dual‐luciferase reporter assays [39, 40], which enables clear mechanistic exploration of the miR‐511‐3p/ATP2A2 axis at the cellular level. Nevertheless, this model carries inherent limitations. H9c2 cells retain cardiomyoblast features and cannot fully recapitulate the mature primary cardiomyocyte electrophysiological contractile phenotype [41]; additionally, this simplified monocellular system lacks the complex multicellular microenvironment, hemodynamic regulation, neurohumoral signaling, and immune cell crosstalk existing in intact myocardium [37]. Therefore, findings obtained from the H9c2‐based H/R model are only valid at the cellular level. The regulatory role of the miR‐511‐3p/ATP2A2 axis and the cardioprotective effect of PPF still require further validation in primary cardiomyocytes and in vivo MIRI animal models to confirm translational potential.

Nevertheless, this study contained several limitations. All experiments were performed exclusively in H9c2 cardiomyocytes under H/R conditions, without validation using in vivo MIRI animal models or human clinical specimens. The rat or mouse left anterior descending (LAD) artery ligation model is the gold‐standard in vivo model for MIRI research [42], which we did not employ here. Moreover, we did not assess potential off‐target effects of miR‐511‐3p manipulation; miRNAs may regulate multiple genes beyond ATP2A2. Additionally, H9c2 cardiomyoblasts possess immature cardiac phenotypes, representing an inherent drawback of this in vitro monoculture system. The in vitro system cannot recapitulate the complex in vivo milieu, including hemodynamic, systemic inflammatory, and neurohumoral factors. Thus, the observed miR‐511‐3p/ATP2A2 axis effects are restricted to the cellular level and limit translational potential. For future work, we will establish rodent LAD artery ligation MIRI models to verify this axis and PPF‐mediated cardioprotection at the tissue level. We will also identify potential off‐target genes induced by miR‐511‐3p overexpression or inhibition, and collect human samples from MIRI patients to explore clinical relevance and strengthen preclinical evidence.

## 5. Conclusion

The miR‐511‐3p/ATP2A2 signaling axis participated in regulating the progression of H/R injury, and alterations of this pathway correlated with the cardioprotective phenotype induced by PPF treatment.

## Funding

This study was supported by the Chengdu Medical Research Program: A Study on the Effects of Astrocyte Activation on Anesthetic Outcomes in Cardiac Surgery and Its Potential Mechanisms, No. 2023562.

## Conflicts of Interest

The authors declare no conflicts of interest.

## Supporting information


**Supporting Information** Additional supporting information can be found online in the Supporting Information section. Table S1 presents the primer sequences used for RT‐qPCR detection. The table contains primer information for miR‐511‐3p, U6, ATP2A2 and GAPDH. U6 and GAPDH were applied as endogenous reference genes for microRNA and mRNA expression measurement, respectively.

## Data Availability

The data that support the findings of this study are available from the corresponding author upon reasonable request.
