# Supplementary material for: The miR‐511‐3p/ATP2A2 Axis Is Required for Propofol‐Mediated Cardioprotection Against Hypoxia/Reoxygenation Injury
Source: Cardiovasc Ther. 2026 Oct 20;2026:7021184. doi: 10.1155/cdr/7021184 (PMC13682209; doi:10.1155/cdr/7021184)
Supplement: Supplementary file 1 [file CDR-2026-7021184-s001.docx]

**Supplementary Table 1.** Primer sequences used for RT-qPCR.

| Gene name | Forward primer (5′‑3′) | Reverse primer (5′‑3′) |
| --- | --- | --- |
| miR-511-3p | CGCGCAATGTGTAGCAAAAGA | ATCCAGTGCAGGGTCCGAGG |
| U6 | CTCGCTTCGGCAGCACA | AACGCTTCACGAATTTGCGT |
| ATP2A2 | ATCTCCCTGCCTGTGATCCT | CACAAAGGGCCAGGAAATGC |
| GAPDH | GACATGCCGCCTGGAGAAAC | AGCCAGGATGCCCTTTAGT |
